# Amino Acid Derivatives as New Zinc Binding Groups for the Design of Selective Matrix Metalloproteinase Inhibitors

**DOI:** 10.1155/2013/178381

**Published:** 2013-03-10

**Authors:** Mariateresa Giustiniano, Paolo Tortorella, Mariangela Agamennone, Antonella Di Pizio, Armando Rossello, Elisa Nuti, Isabel Gomez-Monterrey, Ettore Novellino, Pietro Campiglia, Ermelinda Vernieri, Marina Sala, Alessia Bertamino, Alfonso Carotenuto

**Affiliations:** ^1^Dipartimento di Chimica Farmaceutica e Tossicologica, Università di Napoli “Federico II,” Via D. Montesano 49, 80131 Napoli, Italy; ^2^Dipartimento di Farmacia, Università degli Studi di Bari “Aldo Moro,” Via Orabona 4, 70125 Bari, Italy; ^3^Dipartimento di Farmacia, Università degli Studi “G. d'Annunzio,” Via dei Vestini 31, 66013 Chieti, Italy; ^4^Dipartimento di Scienze Farmaceutiche, Università di Pisa, Via Bonanno 6, 56126 Pisa, Italy; ^5^Dipartimento di Scienze Farmaceutiche e Biomediche, Università degli Studi di Salerno, Via Ponte don Melillo, 84084 Fisciano, Italy

## Abstract

A number of matrix metalloproteinases (MMPs) are important medicinal targets for conditions ranging from rheumatoid arthritis to cardiomyopathy, periodontal disease, liver cirrhosis, multiple sclerosis, and cancer invasion and metastasis, where they showed to have a dual role, inhibiting or promoting important processes involved in the pathology. MMPs contain a zinc (II) ion in the protein active site. Small-molecule inhibitors of these metalloproteins are designed to bind directly to the active site metal ions. In an effort to devise new approaches to selective inhibitors, in this paper, we describe the synthesis and preliminary biological evaluation of amino acid derivatives as new zinc binding groups (ZBGs). The incorporation of selected metal-binding functions in more complex biphenyl sulfonamide moieties allowed the identification of one compound able to interact selectively with different MMP enzymatic isoforms.

## 1. Introduction

**Figure 1:**
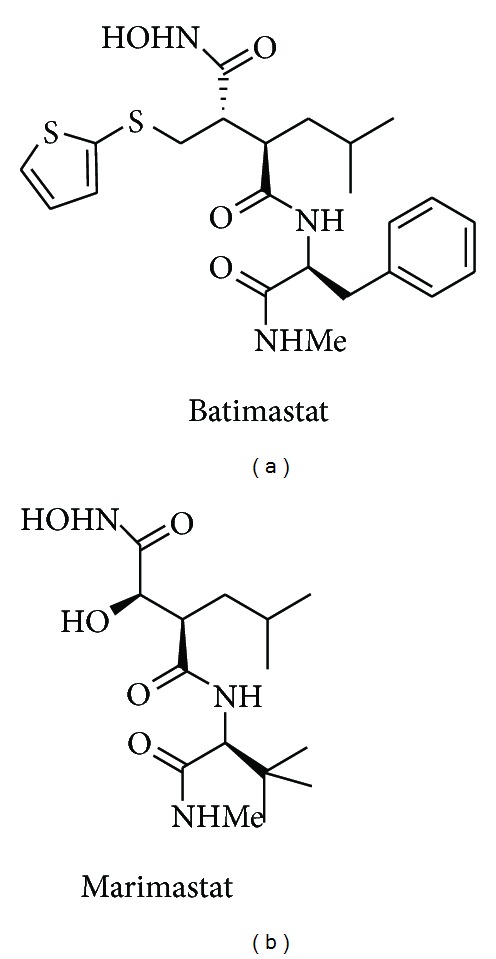
Structures of Batimastat and Marimastat.

 Matrix metalloproteinases (MMPs) are 23-member zinc-dependent endopeptidases family involved in the extracellular matrix turnover [1]. Their aberrant regulation has been implicated in tumoral process, where they showed to have a dual role inhibiting or promoting cell growth and survival, angiogenesis and metastasis [2, 3] differentiation [4], and inflammation and immune surveillance [5]. Moreover, MMPs are overexpressed in a variety of tumor types, and their overexpression is associated with tumor aggressiveness and poor prognosis [6]. The specific alteration of the MMPs in malignant tissues and their participation in some of the major oncogenic mechanisms have both fuelled interest in the design and evaluation of MMP inhibitors (MMPIs) as anticancer agents [7, 8]. Generally, the MMPIs design involves peptide or peptidomimetic backbones containing a zinc-binding group (ZBG) able to interact with both the subpockets surrounding the active site (S_1_ and S_1_′, S_2_′, and S_3_′) and the zinc (II) ion present in the catalytic site, respectively [9, 10]. The greater part of MMPIs research has focused on developing the peptide or peptidomimetic containing a hydroxamic acid as chelating group. Although this design has produced potent inhibitors such as Batimastat [11, 12] and Marimastat [13] (Figure 1), none of these MMPIs has successfully completed clinical trials.

**Figure 2:**
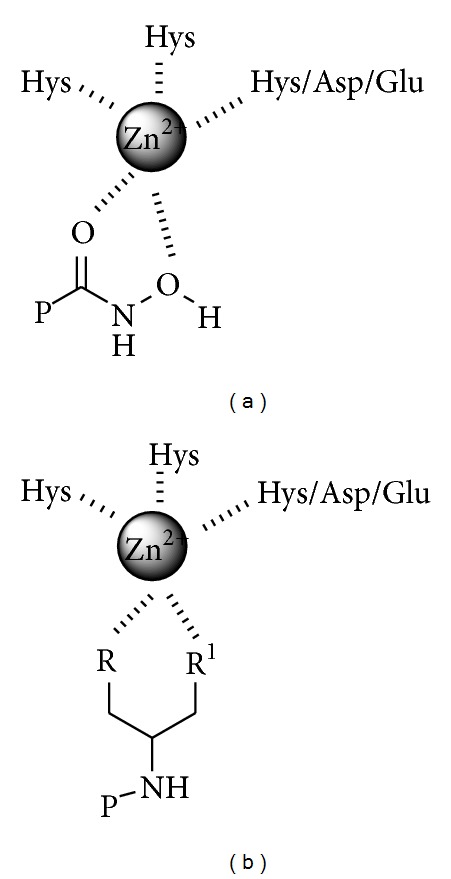
Hypothetical interaction between ZBG and Zn^2+^.

The inability of hydroxamates to produce clinically viable compounds has been attributed to low oral availability, poor in vivo stability, and undesirable side effects associated with these compounds [14]. This has prompted the investigation of a limited number of nonhydroxamate-based MMPIs [15–19]. We present herein the results obtained with a small library of compounds synthesized and tested as potential ZBGs. The compounds were selected on the basis of some similarities to hydroxamates, such as the possibility to form five-member chelates (Figure 2), but with potentially enhanced pharmacokinetic properties such as a better hydrolytic stability and/or proposed increased affinity for the MMP zinc (II).

The designed ligands have a general 2-aminopropane-1,3-disubstituted structure which might be visualized as an amino acid derivative with the *α* carbon atom connected through two *β* carbons to heteroatoms with lone pairs or simply electron availability (R and R_1_). These functional groups are sulfhydryl (SH), alcohol (OH), imidazole, cyano (CN), and azide (N_3_) which are able to interact as Lewis-base in the coordination of the catalytic zinc ion. Their symmetric and asymmetric combination gave rise to a small ZBGs library (Table 1). The two *β* carbons rotational freedom could allow the chelating groups R and R_1_ to orient themselves as better as possible in direction of the zinc ion. 

According to the preliminary results of enzymatic inhibition activities, we further synthesized, from the most interesting ligands, a small series of sulfonamide derivatives containing a phenoxyphenyl group. This moiety has been widely used in the design of MMPs inhibitors as side chain of choice able to interact with the enzymatic S_1_′ subsite which plays a pivotal role in the determination of inhibition selectivity [20, 21]. The aims of the current study were to screen a range of nonhydroxamate structures as new ZBGs and to evaluate the enzymatic activity of small molecules designed to interact with the subpocket S_1_′ and with the zinc (II) ion present in the catalytic site of MMPs.

## 2. Chemistry

**Scheme 1:**
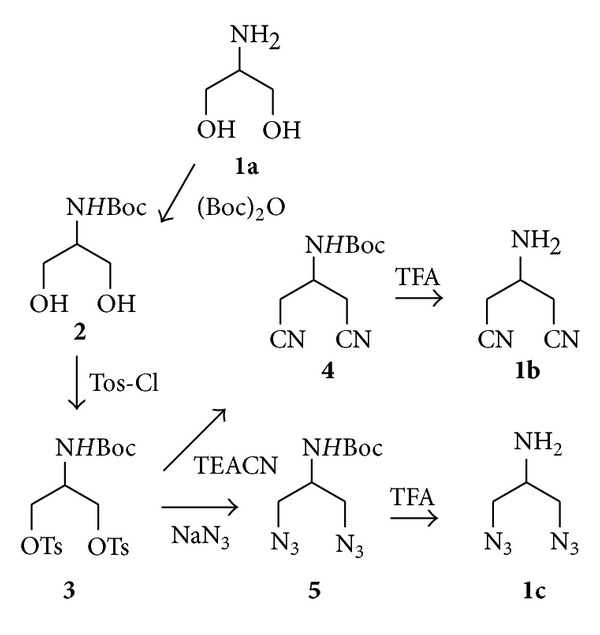
Synthesis of symmetric ZBGs **1a**, **1b**, and **1c**.

The symmetric ligands were prepared starting from serinol (**1a**) according to synthetic route shown in Scheme 1. After N-Boc-protection, the alcohol groups of **2** were activated as ditosylate derivatives in order to undergo nucleophilic substitution with azide and nitrile salts. Thus, displacement of the OTs group with tetraethylammonium cyanide (TEACN) or sodium azide (NaN_3_) in DMF using TEA as base led to **4** and **5**, respectively, with 80%–82% yields. The final symmetric derivatives **1b** and **1c** have been obtained after deprotection of 2-amino group using a solution of 25% TFA in dichloromethane.

The ditosylation reaction was the limiting step in this synthetic strategy, described in the literature using pyridine (py) as solvent [22]. In our case, the treatment of **2** with 4-toluenesulfonyl chloride in pyridine led to ditosylate derivative **3** in only 2% yield. A preliminary study of the influence of solvents, reaction time, and reactive/base concentration ratio on this reaction was performed in order to (a) improve yields and mono/ditosylate adduct ratio; (b) facilitate work-up procedures; (c) use a less toxic solvent.

As shown in Table 2, treatment of **2** with Tos-Cl and TEA in 2.4 : 3 ratio gave the highest yields (85%) and better selectivity (1 : 19) in the formation of ditosylate derivative **3** using dry dichloromethane as solvent (entry 8). Pyridine or pyridine with dimethylaminopyridine as base catalyst gave low yields with a little percentile of dialkylation product (entries 1, 2, and 3), while DCM as solvent was more effective without base catalyst (entries 6, 7, and 8 versus entries 4 and 5).

**Scheme 2:**
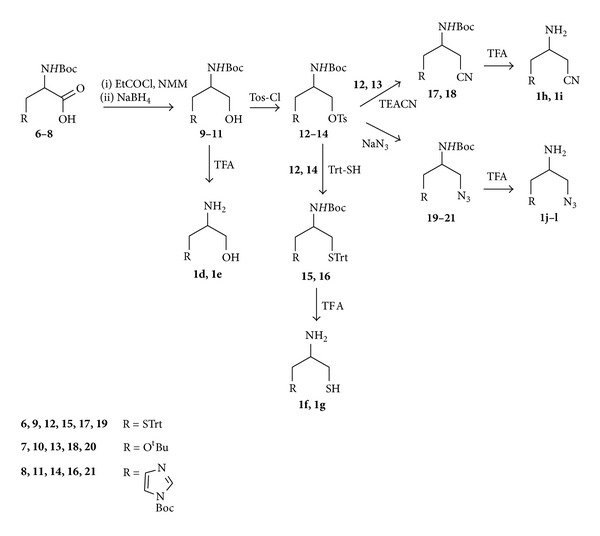
Synthesis of symmetric (**1f**) and asymmetric (**1d, 1e, 1g–1l**) ZBGs.

The symmetric and asymmetric ligands, **1f** and **1d**, **1e**, and **1g–1l**, respectively, were prepared according to the synthetic route shown in Scheme 2. 

Protected amino acids Boc-Cys(Trt)-OH (**6**), Boc-Ser(OtBu)-OH (**7**), and Boc-His(Boc)-OH (**8**) were reduced to corresponding alcohols (**9**–**11**) using sodium borohydride as we previously described [23]. Treatment of hydroxy derivatives **9** and **11** with 20% TFA/DCM gave directly the corresponding final asymmetric ligands **1d** and **1e**. Analogously, reaction of hydroxyl derivatives with Tos-Cl in DCM and TEA led to tosylate intermediates **14**–**16** which were submitted to nucleophilic substitution reaction with azide and nitrile salts in the previously mentioned conditions to give the corresponding cyano (**17**,**18**) and nitrile (**19**–**21**) derivatives. Loss of protective groups after treatment of intermediates **12**–**21** with 50% TFA/DCM solution conduced to final compounds **1f**–**1l**.

**Scheme 3:**
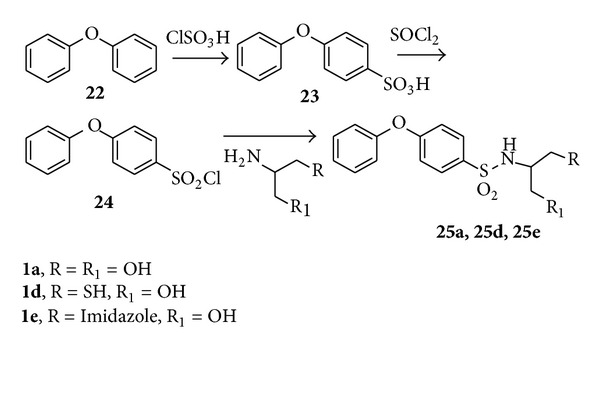
Synthesis of phenoxybenzensulfonamide derivatives **25a**, **25d**, and **25e**.

Finally, the N-substituted phenoxybenzensulfonamide **25a**, **25d**, and **25e** were prepared according to synthetic route of Scheme 3. The sulfonation of diphenylether **22** with chlorosulfonic acid (ClSO_3_H) and afterward chlorination with thionyl chloride (SOCl_2_) afforded the key 4-phenoxybenzensulfonyl chloride **24** with 95% overall yields. The coupling of **24** with **1a**, **1d**, and **1e** in DMF and cesium carbonate (Cs_2_CO_3_) gave directly the corresponding final compounds in 42%–55% yields. 

## 3. Enzymatic Inhibition Assays

The synthesized ZBGs, compounds **1a**–**1l**, were tested against the catalytic domain of MMP-2 in order to evaluate their chelating capability with respect to acetohydroxamic acid (AHA) which was considered a representative of the standard hydroxamate chelator. All the examined compounds exhibited a higher inhibitory activity compared to AHA (Table 3). The enzymatic assays revealed that the most interesting compounds are the serinol **1a** and the asymmetric ligands **1e** and **1g, **containing both an imidazole group and an alcohol or a thiol group, respectively. Surprisingly, the cysteinol **1d**, despite the well-known zinc thiophilicity, showed a lower enzymatic activity with IC_50_ value of 674 *μ*M.

On the basis of these data, we selected the most active ligands **1a** and **1e** to be incorporated as ZBG in a more complex structure. The ligands were linked, through a sulfonamide bind, with a phenoxyphenyl group, described in the literature for its well-validated affinity for the S_1_′ enzymatic subpocket [10, 24]. A third ligand **1d**, less active, was also chosen in order to evaluate the real influence of ZBG group alone in the enzymatic activity.

Compounds **25a**, **25d**, and **25e** were tested against human recombinants MMP-1, MMP-2, MMP-8, and MMP-9 by a fluorometric assay, and the obtained IC_50_ values are summarized in Table 4. Compound **25a** exhibited an interesting inhibitory activity on MMP-2 and MMP-8, two enzymatic isoforms characterized by an intermediate and a deep S_1_′ subpocket, respectively [25–28]. This compound showed also a good selectivity over MMP-1 which has a shallow S_1_′ pocket. The substitution of a hydroxyl with an imidazole group, compound **25e**, caused a loss of both potency (except on MMP-1) and selectivity on the enzymes used in this study. The most interesting results were obtained with compound **25d**. This compound showed a high inhibitory activity on MMP-8 and MMP-9 with IC_50_ values in the micromolar range (10- and 13-fold more potent than **25a**, resp.) and maintained a good selectivity over both MMP-2 and MMP-1. 

These preliminary results showed a different behaviour of ZBGs when they are introduced into a more complex structure indicating that, in this case, the modulation of selectivity does not depend only on ZBGs [29].

## 4. Molecular Modeling

In order to rationalize the observed activity data, docking calculations of the ZBGs and compounds **25a**, **25d**, and **25e** were performed on the MMP-2 catalytic domain. Subsequently, they were submitted to a refinement step, thorough minimization of best poses. The applied protocol allowed to correlate predicted and experimental binding energies. It is well known that docking scores hardly correlate with activity data, and to this aim, more accurate calculations are required such as Free Energy Perturbation or Thermodynamic Integration. Among available approaches, Linear Interaction Energy (LIE) represents a good compromise between accuracy and speed of calculations [30, 31]. In this approach, the binding process is represented as the replacement of water molecules solvating a ligand by the protein, using an implicit water model.

LIE generates a custom scoring function calculating the values of alpha, beta, and gamma coefficients of the following equation:

(1) Delta  G=alpha∗(U  vdw_b−U  vdw_f)+beta∗(U  elec_b−U  elec_f)+gamma∗(U  cav_b−U  cav_f),

where Delta *G* is the calculated binding energy; *U*xxx_*b* is the van der Waals, Coulombic, and Cavity energy terms from the bound state; *U*xxx_*f* is the van der Waals, Coulombic, and Cavity energy terms from the free state.

LIE method applied to our ligands provided a statistically significant correlation between calculated and experimental data, underpinning the validity of predicted docking poses (Table 5). 

It is worth noting that chiral compounds under study were synthesized and tested as racemic mixture. Consequently, all calculations were carried out for all enantiomers, and quantitative models were generated for both R (R-model) and S forms (S-model) separately. Obtained Δ*G* values indicate that the R-model works slightly better than the S-model in predicting activity, as demonstrated by statistical correlation values (Table 6); however, the S-model is able to predict the binding energy with acceptable approximation indicating that experimental activity can be due to the contribution of both enantiomers.

This result is confirmed from the analysis of fragments docking poses in fact that no relevant differences can be observed in the binding of enantiomeric forms of chiral compounds, in the MMP-2 active site.

**Figure 3:**
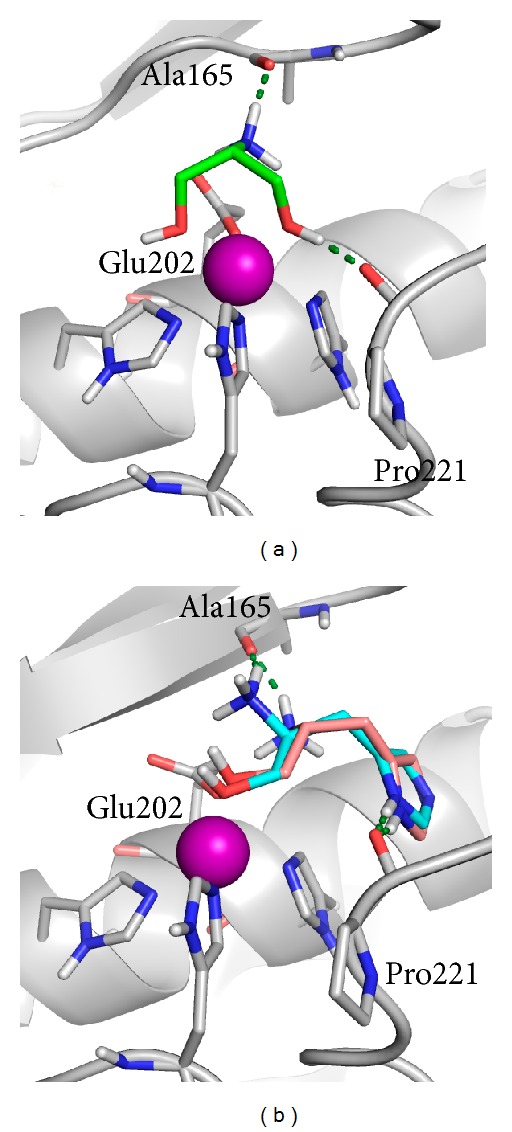
Docked poses of **1a** (a) and **1e** in both enantiomeric forms (b) into the MMP-2 active site. MMP-2 is represented as a grey cartoon. Ligands and most relevant residues are depicted as sticks. H-bonds are represented as green dashed lines.

Moreover, differently than expected, just ligand **1a** is able to chelate the zinc ion, providing an explanation of the higher activity observed for this compound. Other fragments give a monodentate binding of the catalytic zinc, and the other electron donating group is usually involved in H-bond interactions with surrounding residues, such as the Pro221 carbonyl oxygen (e.g., **1d**), except for compounds containing the imidazole ring (e.g., **1e**), involved in a *π*-*π* stacking with the His201 side chain, which represents one of the main interactions formed by MMPIs in the S_1_′ pocket (Figure 3). This behavior can be attributed to the strict geometrical requirements, which must be fulfilled by chelating group around the zinc ion in MMPs active site. 

The binding of sulfonamide derivatives **25a**, **25d**, and **25e** was studied as well through docking calculation and subsequent refinement as previously described on MMP-1, -8, and -9 (Table 7). No statistical correlations are provided in these cases because of the few available data. Docking results show all ligands occupying the S_1_′ site, except for MMP-1. This isoform, in fact, is known for having a short S_1_′ pocket, unable to accommodate the large biphenylether portion of these ligands. The imidazole ring of compounds **25e**, the more active towards MMP-1, occupy the hydrophobic pocket of this protein. 

**Figure 4:**
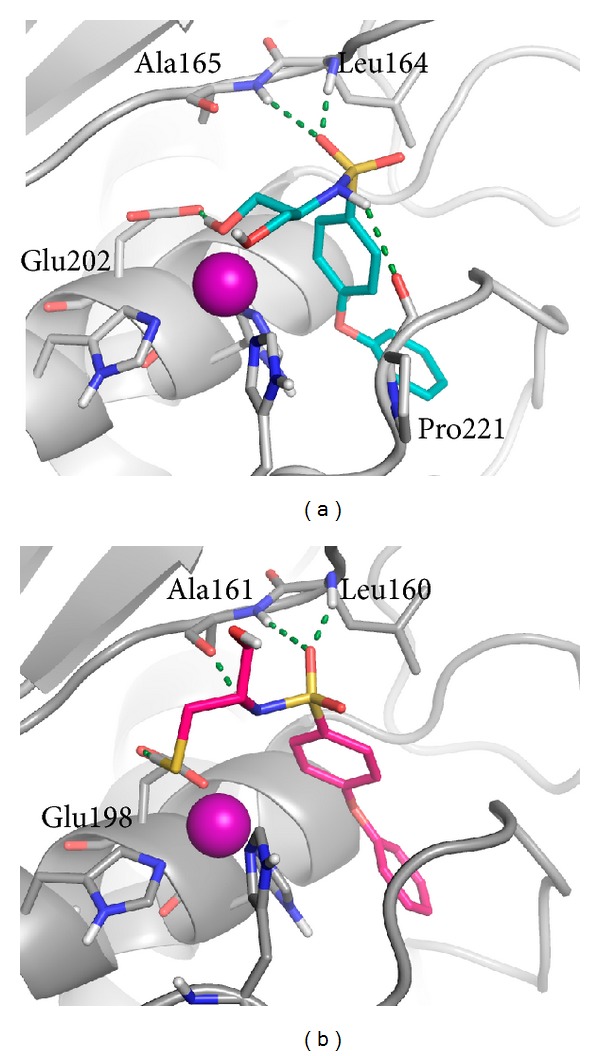
Docked poses of **25a** into the MMP-2 active site (a) and **25d** into MMP-8 active site (b). MMP-2 and MMP-8 are represented, respectively, as a grey and dark grey cartoons. Ligands and most relevant residues are depicted as sticks. H-bonds are represented as green dashed lines.

Binding mode of sulfonamide derivatives to the other MMPs is well conserved, regardless of chirality: MMP-2, -8, and -9 have a deep S_1_′ site able to locate the hydrophobic biphenyl ether, whose proximal aromatic ring interacts with the imidazole ring of His201, and the distal ring provides hydrophobic interactions in binding pocket. The sulfonamide moiety provides two H-bonds between a sulfone oxygen and Ala165 and Leu164 NH (MMP-2 numbering) and the sulfonamide NH and the Pro221 CO or alternatively Ala165 CO (Figure 4). Main differences are observed for the binding of the ZBG; in MMP-2, the ZBG of **25a** maintains the ability to chelate the zinc ion.

This chelation, not observed in MMP-8 and -9, can explain the higher activity observed for this ligand in MMP-2.

MMP-8 and -9 zinc ions coordinate all ligands in a monodentate fashion with a similar geometry, similarly to what observed for the ZBG in MMP-2. Therefore, as no chelation is provided by the ZBG in MMP-8 and MMP-9, the zinc thiophilicity seems to play a relevant role in determining activity toward these isoforms. 

## 5. Conclusion

Herein, we described the design, synthesis, inhibitory activity, and molecular modeling studies of new non-hydroxamate-based MMPIs. The adopted synthetic strategy enabled the setting-up of a small ZBGs library through a simple and easily accessible pool of reactions. The biological screening of this library led to the identification of two ZBGs that were incorporated in a more complex structure able to interact with the S_1_′ enzymatic site. The biological data for compounds **25a** and **25e** confirmed the inhibition trend of the respective ZBGs against MMP-2. Compound **25d**, containing a less potent chelating group (**1d**  versus **1a** and **1e**), was equipotent to **25a** against MMP-2 and more potent than **25a **against MMP-8 and MMP-9 (10- and 13-fold, resp.). Molecular modeling studies provided a rationalization of the experimental data, suggesting a putative binding mode of studied ligands in MMPs active site. These preliminary results indicate the importance of testing and selecting firstly compounds containing the minimums structural requirements necessary for a specific biological activity. Furthermore, taking in consideration the complex role of MMPs in the cellular and tumoral homeostasis, the development of selective inhibitors is desirable in order to shed further light on the protein function, signalling pathways, and role in disease of different MMPs [32–34]. Thus, compound **25d** identified in this preliminary study as MMP-8 and MMP-9 inhibitors could be submitted to a rational process of hit optimization with the aim to improve its potency and selectivity of action. The introduction of these new fragments into different peptide structures with the aim to synthesize selective MMPs inhibitors and to explore their structure-activity relationships is currently under study in our laboratory.

## 6. Experimental 

### 6.1. MMP Inhibition Assays

Pro-MMP-1, pro-MMP-2, pro-MMP-8, and pro-MMP-9 were purchased from Calbiochem. Proenzymes were activated immediately prior to use with *p*-aminophenylmercuric acetate (APMA 2 mM for 1 h at 37°C for MMP-2 and MMP-8, APMA 2 mM for 2 h at 37°C for MMP-1, and APMA 1 mM for 1 h at 37°C for MMP-9). For assay measurements, the inhibitor stock solutions (10 mM in DMSO) were further diluted, at seven different concentrations (0.01 nM–200 *μ*M) for each MMP in the fluorometric assay buffer (FAB: Tris 50 mM, pH = 7.5, NaCl 150 mM, CaCl_2_ 10 mM, Brij 35 0.05%, and DMSO 1%). Activated enzyme (final concentration 0.56 nM for MMP-2, 1.3 nM for MMP-9, 1.5 nM for MMP-8, and 2.0 nM for MMP-1) and inhibitor solutions were incubated in the assay buffer for 4 h at 25°C. After the addition of 20 *μ*M solution of the fluorogenic substrate Mca-Lys-Pro-Leu-Gly-Leu-Dap(Dnp)-Ala-Arg-NH_2_ (Bachem) for all the enzymes in DMSO (final concentration 2 *μ*M), the hydrolysis was monitored every 15 s for 15 min recording the increase in fluorescence (*λ*ex = 325 nm; *λ*em = 395 nm) using a Molecular Devices SpectraMax Gemini XS plate reader. The assays were performed in triplicate in a total volume of 200 *μ*L per well in 96-well microtiter plates (Corning, black, NBS). The MMP inhibition activity was expressed in relative fluorescent units (RFUs). Percent of inhibition was calculated from control reactions without the inhibitor. IC_50_ was determined using the formula: *V*_*i*_/*V*_*o*_ = 1/(1 + [I]/IC_50_), where *V*_*i*_ is the initial velocity of substrate cleavage in the presence of the inhibitor at concentration [I], and *V*_*o*_ is the initial velocity in the absence of the inhibitor. Results were analyzed using SoftMax Pro software and Origin software.

### 6.2. General

Reagents, starting materials, and solvents were purchased from commercial suppliers and used as received. Analytical TLC was performed on plates coated with a 0.25 mm layer of silica gel 60 F254 Merck and preparative TLC on 20 cm × 20 cm glass plates coated with a 0.5 mm layer of silica gel PF254Merck. Silica gel 60 (300–400 mesh, Merck) was used for flash chromatography. Melting points were determined by a Kofler apparatus and are uncorrected. ^1^H NMR and ^13^C NMR spectra were recorded with a Varian-400 spectrometer, operating at 400 and 100 MHz, respectively. Chemical shifts are reported in *δ* values (ppm) relative to internal Me_4_Si, and *J* values are reported in hertz (Hz). ESIMS experiments were performed on an Applied Biosystems API 2000 triple-quadrupole spectrometer. 

#### 6.2.1. 2-(tert-Butyloxycarbonyl)-1,3-dihydroxypropane **(2)**

To a 25 mL round-bottom flask, 2-aminopropane-1,3-diol **1a** (11 mmol) (Sigma-Aldrich, 98%) was added and dissolved in a 1 : 1 mixture water/1,4-dioxan (10 mL). After few minutes, di-tert-butyl dicarbonate (1.2 eq) and KOH until pH 8 were added. The reaction was stirred for 48 h, washed with H_3_O^+^, dried with Na_2_SO_4_, and evaporated under reduced pressure (yield: 98%); ^1^H-NMR (400 MHz, CDCl_3_) *δ* 1.34 (s, 9H, Boc); *δ* 3.59–3.63 (m, 1H, H-2); *δ* 3.72–3.87 (m, 4H, H-1 and H-3); *δ* 5.15 (bs, 1H, N*H*Boc)). 

#### 6.2.2. 2-(tert-Butoxycarbonylamino)propane-1,3-diyl bis(4-Methylbenzenesulfonate) **(3)**

To a 25 mL round-bottom flask, **2** (10 mmol) was added and dissolved in dry DCM (10 mL). After reached 0°C, paratoluensulfonyl chloride (2.4 eq) and TEA (3 eq) were added. The reaction was stirred for 10 h, washed with water, dried with Na_2_SO_4_, and evaporated under reduced pressure. The crude was then purified by chromatographic column using *n*-hexane/AcOEt 2 : 1 as eluent (yield: 85%); ^1^H-NMR (400 MHz, CDCl_3_) *δ* 1.34 (s, 9H, Boc); 4.00–4.06(m, 5H, H-1, H-2, and H-3); 4.89 (bs, N*H*Boc); 7.31 (d, *J* = 8 Hz, 4H, aryl); 7.71 (d, 4H, aryl).

## 7. General Procedure for the Synthesis of Symmetric Ligands 3-Aminopentanedinitrile (1b) and 1,3-Diazidopropan-2-amine (1c)

To a 25 mL round-bottom flask, **3** (5 mmol) was added and dissolved in DMF (10 mL). TEA (3 eq) and TEACN (2.4 eq) or NaN_3_ (2.4 eq) were then added, and the reaction was stirred for 10 h at room temperature. The reaction mixtures were washed with water, dried with Na_2_SO_4_, and evaporated under reduced pressure. The crudes **4** and **5** were purified by chromatographic column using *n*-hexane/AcOEt: 3/1 as eluent. Data for *tert*-butyl 1,3-dicyanopropan-2-ylcarbamate (**4**), Data for *tert*-butyl 1,3-dicyanopropan-2-ylcarbamate (**4**) 1H-NMR(400 MHz, CDCl3): *δ* 1.41 (s, 9H); 2.73–2.82 (m, 4H,H-2 and H-4); 3.47–3.50 (m, 1H, H-3); 5.06 (bs, 1H, N*H*Boc). Data for *tert*-butyl 1,3-diazidopropan-2-ylcarbamate (**5) **1H-NMR (400 MHz, CDCl3): *δ* 1.49 (s, 9H, Boc); 3.40–3.52 (m, 4H, H-1 and H-3); 3.86–3.90 (m,1H, H-2); 4.77 (bs, 1H,N*H*Boc). A solution of derivatives **4** or **5** (1 mmol) in CH_2_Cl_2_ (10 mL) was treated with trifluoroacetic acid (10 mL) and stirred at room temperature. The reaction was stirred for 2 h at room temperature and evaporated under reduced pressure to yield the corresponding final products as TFA salt.

### 7.1. 3-Aminopentanedinitrile Trifluoroacetate **(1b)**

Yield: 36%. ^1^H-NMR (400 MHz, CD_3_OD) *δ* 3.00–3.03 (m, 4H, H-2 and H-4); 3.07 (t, 1H, *J* = 6.0 Hz). ^13^C-NMR (100 MHz, CD_3_OD) *δ* 20.7 (C-3, C-4), 39.5 (C-3), 114.9 (CN). ESI-MS calc for C_15_H_17_NO_5_S 323.08, found 323.16. 

### 7.2. 1, 3-Diazidopropan-2-amine Trifluoroacetate **(1c)**

Yield: 33%.  ^1^H-NMR (400 MHz, CD_3_OD) *δ* 3.46–3.48 (m, 1H, H-2); 3.63–3.77 (m, 4H, H-1, H-3). ^13^C-NMR (100 MHz, CD_3_OD) *δ* 52.0 (C-2), 59.0 (C-1 and C-3). ESI-MS calc for C_15_H_17_NO_5_S 323.08, found 323.16. 

## 8. General Procedure for Synthesis of Amino Alcohols Derived from Amino Acids (9–11)

 Ethyl chloroformate (1.2 eq) and N-methylmorfoline (1.2 eq) at 0°C were added to a solution of Boc-Cys(Trt)-OH (**6**) or Boc-Ser(OtBu)-OH (**7**) or flask Boc-His(Boc)-OH (**8**) (1 mmol) in THF (4 mL). After 1 h, the reaction was filtered off, and NaBH_4_ (3 eq) dissolved in 2 mL of water was added. The reaction was then stirred at room temperature for 3 h, washed with H_3_O^+^, dried with Na_2_SO_4_, and evaporated under reduced pressure. Chromatography purification of the corresponding residues using *n*-hexane/AcOEt: 2/1 yielded, in each case, the amino alcohol derivatives.

### 8.1. tert-Butyl 1-Hydroxy-3-(tritylthio)propan-2-ylcarbamate **(9)**

Yield: 73%. ^1^H-NMR (400 MHz, CDCl_3_) *δ* 1.38 (s, 9H, Boc); 2.40–2.42 (m, 2H, H-3); 3.46–3.51 (m, 3H, H-1, H-2); 4.77 (bs, 1H, N*H*Boc); 7.20–7.44 (m, 15H, aryl).

### 8.2. tert-Butyl 1-(tert-Butoxy)-3-hydroxypropan-2-ylcarbamate (10)

Yield: 69%. ^1^H-NMR (400 MHz, CDCl_3_) *δ* 1.08 (s, 9H); 1.41 (s, 9H); 3.12 (m, 1H, H-3); 3.41–3.49 (m, 2H, H-1, H-3); 3.62 (m, 2H, H-1, H-2); 5.12 (s, N*H*Boc). 

### 8.3. tert-Butyl 1-Hydroxy-3-(1H-imidazol-4-yl)propan-2-ylcarbamate **(11)**

Yield: 80%. ^1^H-NMR (400 MHz, CDCl_3_) *δ* 1.39 (s, 9H); 1.45 (s, 9H); 2.61–2.72 (m, 2H, H-1); 3.12–3.21 (m, 2H, H-3); 3.62 (m, 1H, H-2); 4.98 (bs, N*H*Boc); 7.00 (s, 1H, imidazole); 8.21(s, 1H, imidazole). 

## 9. General Procedure for Removal of the Boc Protecting Group: Synthesis of Final Ligands 1d and 1e

The compounds **9** or **11** were dissolved in a 1 : 1 mixture DCM/TFA (10 mL), adding triethylsilane (0.1 eq) as scavenger. The reaction was stirred for 2 h at room temperature and evaporated under reduced pressure to yield the title derivatives as TFA salt.

### 9.1. 2-Amino-3-mercaptopropan-1-ol Trifluoroacetate **(1d)**

Amorphous solid (46%). ^1^H-NMR (400 MHz, D_2_O) *δ* 2.45–2.49 (m, 2H, H-3); 3.15–3.19 (m, 1H, H-2); 3.67–3.71 (m, 2H, H-1). ^13^C NMR (100 MHz, D_2_O) *δ* 30.2 (C-3) 57.2 (C-2), 63.1 (C-1). ESI-MS calc for C_5_H_10_F_3_NO_3_S 221.20, found 221.29. 

### 9.2. 2-Amino-3-(1H-imidazol-4-yl)propan-1-ol Ditrifluoroacetate **(1e)**

White solid (39%), m.p. 218–220°C. ^1^H NMR (400 MHz, CD_3_OD) *δ* 2.90–2.93 (m, 2H, H-3); 3.08–3.12 (m, 1H, H-1); 3.66–3.71 (m, 2H, H-1); 7.01 (s, 1H, imidazole); 7.89 (s, 1H, imidazole). ^13^H-NMR (100 MHz, CD_3_OD) *δ* 29.4 (C-3), 58.2 (C-2), 64.1 (C-1) 118.2, 130.1, 134.7 (imidazole). ESI-MS calc for C_10_H_13_F_6_N_3_O_5_ 369.22, found 369.16. 

## 10. General Procedure for Synthesis of Tosilated Derivatives 12–14

To a 25 mL round-bottom flask, **9**, **10**, or **11** (1.1 mmol) was added and dissolved in dry DCM (10 mL). After reached 0°C, paratoluensulfonyl chloride (1.2 eq) and TEA (1.5 eq) were added. The reaction is stirred for 10 h, washed with water, dried with Na_2_SO_4_, and evaporated under reduced pressure. The crudes were then purified by chromatographic column using *n*-hexane/AcOEt: 3/1 as elution system. 

### 10.1. 2-(tert-Butoxycarbonylamino)-3-(tritylthio)propyl 4-Methylbenzenesulfonate **(12)**

Yield: 73%.  ^1^H-NMR (400 MHz, CDCl_3_) *δ* 1.43 (s, 9H); 2.30 (s, 3H, CH_3_); 2.33–2.42 (m, 2H, H-3); 3.55–3.58 (m, 1H, H-1); 3.89–3.93 (m, 3H, H-1, H-2), 4.48 (bs, N*H*Boc); 7.21–7.37 (m, 17 H, aryl); 7.72 (d, *J* = 8.0 Hz, 2H, aryl).

### 10.2. 3-tert-Butoxy-2-(tert-butoxycarbonylamino)propyl 4-Methylbenzenesulfonate **(13)**

Yield: 77%.  ^1^H-NMR (400 MHz, CDCl_3_) *δ* 1.08 (s, 9H); *δ* 1.43 (s, 9H); 2.31 (s, 3H, CH_3_); 3.14–3.23 (m, 2H, H-1, H-3); 3.87–3.91 (m, 2H, H-1, H-3); 4.02–4.08 (m, 1H, H-2); 4.92 (bs, N*H*Boc); 7.16 (d, *J* = 8.1 Hz, 2H, aryl); 7.89 (d, 2H, aryl).

### 10.3. tert-Butyl 4-(2-(tert-Butoxycarbonylamino)-3-(tosyloxy)propyl)-1H-imidazole-1-carboxylate **(14)**

Yield: 73%.  ^1^H-NMR (400 MHz, CDCl_3_) *δ* 1.38 (s, 9H); 1.41 (s, 9H); 2.31 (s, 3H, CH_3_); 2.59–2.65 (m, 2H, H-1); 3.53–3.56 (m, 1H, H-3); 3.74–3.82 (m, 1H, H-3); 4.00–4.07 (m, 1H, H-2); *δ* 5.01 (bs, N*H*Boc); 7.01 (s, 1H, imidazole); 7.19 (d, *J* = 8.0 Hz, 2H, aryl); 7.80 (d, 2H, aryl); 7.89 (s, 1H, imidazole).

## 11. General Procedure for the Synthesis of Thio Derivatives 15 and 16

To a 25 mL round-bottom flask, **12** or **14** (1.1 mmol) was added, and dissolved in DMF (10 mL). TEA (1.5 eq) and Trt-SH (1.2 eq) were then added and the reaction was stirred for 10 h at room temperature. The reaction mixture was then washed with water, dried with Na_2_SO_4_, and evaporated under reduced pressure. The crudes were then purified by chromatographic column using TLC: *n*-hexane/AcOEt: 4/1 as eluent system.

### 11.1. tert-Butyl 1,3-bis(Tritylthio)propan-2-ylcarbamate **(15)**

Yield: 81%. ^1^H-NMR (400 MHz, CDCl_3_) *δ* 1.39 (s, 9H); 2.38–2.41 (m, 4H, H-1, H-3); 4.01–4.05 (m, 1H, H-2); 4.48 (bs, N*H*Boc); 7.08–7.23 (m, 30H, aryl). 

### 11.2. tert-Butyl 4-(2-(tert-Butoxycarbonylamino)-3-(tritylthio)propyl)-1H-imidazole-1-carboxylate **(16)**

Yield: 79%. ^1^H-NMR (400 MHz, CDCl_3_) *δ* 1.42 (s, 9H); 2.51–2.64 (m, 4H, H-1, H-3); 4.20–4.26 (m, 1H, H-2); 4.48 (bs, N*H*Boc); 7.08–7.23 (m, 16H, aryl); 7.91 (s, 1H, imidazole). 

## 12. Synthesis of Final Ligands 1f and 1g

The compounds **15** or **16** were dissolved in a 1 : 1 mixture DCM/TFA (10 mL), adding triethylsilane (0.1 eq) as scavenger. The reaction was stirred for 2 h at room temperature and evaporated under reduced pressure to yield the title derivatives as TFA salt. 

### 12.1. 2-Aminopropane-1,3-dithiol Trifluoroacetate **(1f)**

Amorphous solid (59%). ^1^H NMR (400 MHz, D_2_O) *δ* 2.64 (dd, 2H, *J* = 6.8 and 11.2 Hz, H-1, H-3); 2.74 (dd, *J* = 5.2 and 6.9 Hz, 2H, H-1, H-3). ^13^C NMR (100 MHz, D_2_O) *δ* 30.1 (C-1, C-3), 55.0 (C-2). ESI-MS calc for C_5_H_10_F_3_NO_2_S_2_: 237.01; found 237.11. 

### 12.2. 2-Amino-3-(1H-imidazol-2-yl)propane-1-thiol Ditrifluoroacetate **(1g)**

White solid (61%), m.p. 196–198°C. ^1^H NMR (400 MHz, CD_3_OD) *δ* 2.79 (dd, 1H, *J* = 5.7 and 10.1 Hz, H-1); 2.90 (dd, 1H, H-1); 3.18–3.25 (m, 2H, H-3); 3.61–3.70 (m, 1H. H-2). ^13^H-NMR (100 MHz, CD_3_OD) *δ* 25.8 (C-1); 26.4 (C-3), 52.3 (C-2), 118.2 (imidazole), 134.7 (imidazole). ESI-MS calc for C_8_H_12_F_3_N_3_O_2_S: 271.06; found 271.10. 

## 13. General Procedure for the Synthesis of Cyano Derivatives 17 and 18

To a 25 mL round-bottom flask, **12** or **13** (1.1 mmol) was added and dissolved in DMF (10 mL). TEA (1.5 eq) and TEACN (1.2 eq) were added, and the reaction was stirred for 10 h at room temperature. The reaction mixtures were then washed with water, dried with Na_2_SO_4_, and evaporated under reduced pressure. The crudes were purified by chromatographic column using *n*-hexane/AcOEt: 3/1 

### 13.1. tert-Butyl 1-Cyano-3-(tritylthio)propan-2-ylcarbamate **(17)**

Yield: 84%. ^1^H-NMR (400 MHz, CDCl_3_) *δ* 1.42 (s, 9H,); 2.29–2.33 (m, 2H, H-3); 2.71–2.86 8 m, 2H, H-1); 3.86–3.91 (m, 1H, C-2); 4.97 (bs, N*H*Boc); *δ* 7.08–7.45 (m, 15H, aryl).

### 13.2. tert-Butyl 1-tert-Butoxy-3-cyanopropan-2-ylcarbamate **(18)**

Yield: 81%. ^1^H-NMR (400 MHz, CDCl_3_) *δ* 1.12 (s, 9H); 1.41 (s, 9H); 2.69 (m, 2H, H-3); 3.23–3.41 (m, 2H, H-1); 3.90–3.94 (m, 1H, H-2); 4.99 (bs, N*H*Boc).

## 14. Synthesis of Final Ligands 1h and 1i

The intermediates **17** and **18** were dissolved in a 1 : 1 mixture DCM/TFA (10 mL), adding triethylsilane (0.1 eq) as scavenger. The reaction was stirred for 2 h at room temperature and evaporated under reduced pressure to afford the title compounds as TFA salt.

### 14.1. 3-Amino-4-mercaptobutanenitrile Trifluoroacetate **(1h)**

Amorphous solid (65%). ^1^H NMR (400 MHz, CD_3_OD) *δ* 3.09–3.20 (m, 4H, H-2, H-4); 3.87–3.90 (m, 1H, H-3). ^13^C NMR (100 MHz, CD_3_OD) *δ* 20.3 (C-2), 38.3 (C-4), 46.1 (C-3), 115.4 (C-1). ESI-MS calc for C_6_H_9_F_3_N_2_O_2_S 230.01, found 230.12.

### 14.2. 3-Amino-4-hydroxybutanenitrile Trifluoroacetate **(1i)**

White solid (63%), m.p. 131–133°C. ^1^H-NMR (400 MHz, CD_3_OD) *δ* 2.41–2.53 (m, 2H, H-2); 3.76–3.89 (m, 3H, H-3, H-4). ^13^C NMR (100 MHz, CD_3_OD) *δ* 21.1 (C-2), 50.0 (C-3), 61.3 (C-4), 114.7 (C-1) ESI-MS calc for C_6_H_9_F_3_N_2_O_3_ 214.06, found 214.16.

## 15. General Procedure for the Synthesis of Azido Derivatives 19–21

To a 25 mL round-bottom flask, **12**, **13**, or **14** (1.1 mmol) were added and dissolved in DMF (10 mL). TEA (3 eq) and NaN_3_ (2.4 eq) were added, and the reactions were stirred for 10 h at room temperature. The reaction mixtures were washed with water, dried with Na_2_SO_4_, and evaporated under reduced pressure. The crudes were then purified by chromatographic column using *n*-hexane/AcOEt: 3/1 as eluent system. 

### 15.1. tert-Butyl 1-Azido-3-(tritylthio)propan-2-ylcarbamate **(19)**

Yield: 75%. ^1^H-NMR (400 MHz, CDCl_3_) *δ* 1.49 (s, 9H); 2.54–2.62 (m, 2H, H-3); 3.29–3.35 (m, 2H, H-1); 3.88–3.90 (m, 1H, H-2); 4.82 (bs, N*H*Boc).

### 15.2. tert-Butyl 1-Azido-3-tert-butoxypropan-2-ylcarbamate **(20)**

Yield: 72%. ^1^H-NMR (400 MHz, CDCl_3_) *δ* 1.18 (s, 9H); 1.43 (s, 9H); 3.01–3.23 (m, 2H, H-1); 3.81–3.90 (m, 3H, H-2, H-3); 4.91 (bs, N*H*Boc).

### 15.3. tert-Butyl 4-{3-Azido-2-[(tert-butoxycarbonyl)amino]propyl}-1H-imidazole-1-carboxylate **(21)**

Yield: 70%.  ^1^H-NMR (400 MHz, CDCl_3_) *δ* 1.39 (s, 9H); 1.59 (s, 9H); 3.21–3.33 (m, 4H, H-1, H-3); 3.98–4.03 (m, 1H, C-2); 4.50 (bs, N*H*Boc). 

## 16. Synthesis of Final Derivatives 1j–1l 

The intermediates **19**, **20**, and **21** were dissolved in a 1 : 1 mixture DCM/TFA (10 mL), adding triethylsilane (0.1 eq) as scavenger. The reaction was stirred for 2 h at room temperature and evaporated under reduced pressure to afford the title compounds as TFA salt.

### 16.1. 2-Amino-3-azidopropane-1-thiol Trifluoroacetate **(1j)**

Amorphous solid (38%). ^1^H-NMR (400 MHz, D_2_O) *δ* 2.76–2.82 (m, 2H, H-3); 3.58–3.62 (m, 2H, H-2, H-1); 3.78–3.81 (m, 1H, H-1). ^13^C NMR (100 MHz, D_2_O) *δ* 30.6 (C-1), 51.8 (C-2), 58.9 (C-3). ESI-MS calc for C_5_H_9_F_3_N_4_O_2_S 264.04, found 264.12

### 16.2. 2-Amino-3-azidopropane-1-ol Trifluoroacetate **(1k)**

Amorphous solid (35%). ^1^H-NMR (400 MHz, D_2_O) *δ* 3.30–3.33 (m, 1H, H-2); 3.40–3.46 (m, 2H, H-3); 3.53–3.64 (m, 2H, H-1). ^13^C NMR (100 MHz, D_2_O) *δ* 50.2 (C-3), 52.0 (C-2), 59.1 (C-1). ESI-MS calc for C_5_H_9_F_3_N_4_O_3_ 230.15, found 230.27.

### 16.3. 1-Azido-3-(1H-imidazol-4-yl)propan-2-amine Ditrifluoroacetate **(1l)**

Amorphous solid (41%). ^1^H-NMR (400 MHz, CD_3_OD) *δ* 3.12–3.15 (m, 2H, H-1); 3.62–3.66 (m, 1H, H-2); 3.62–3.66 (m, 1H, H-2); 3.70–3.83 (m, 2H, H-3); 7.48 (s, 1H, imidazole); 8.86 (s, 1H, imidazole). ^13^C NMR (100 MHz, CD_3_OD) *δ* 25.3 (C-3), 49.7 (C-2), 51.2 (C-1), 118.1, 128.1,134.7(C-imidazole). ESI-MS calc for C_10_H_12_F_6_N_6_O_4_ 394.23, found 394.31.

### 16.4. 4-Phenoxybenzene-1-sulfonyl Chloride **(24)**

In a 25 mL round-bottom flask, **22** (11.75 mmol) was dissolved in dry DCM (10 mL), and chlorosulphonic acid (11.75 mmol.) was added at 0°C. The reaction was stirred for 2 h, evaporated under vacuum, and used for next step without further purification. The reaction mixture was indeed dissolved in thionyl chloride at 0°C and refluxed for 5 h to yield after evaporation product **24** with 95% yield. ^1^H-NMR (400 MHz, CDCl_3_) *δ* 7.01–7.14 8 m, 5H, aryl); 7.41 (d, *J* = 8.6 Hz, 2H, aryl); 7.82 (d, 2H, aryl).

### 16.5. N-(1,3-Dihydroxypropan-2-yl)-4-phenoxybenzenesulfonamide **(25a)**

To a 25 mL round-bottom flask, **1a** (3 mmol) was added and dissolved in acetone (10 mL). NaHCO_3_ (1.5 eq.) and **24** (1.2 eq.) were added, and the reaction was stirred for 24 h at room temperature. The reaction mixture was then washed with water, dried with Na_2_SO_4_, and evaporated under reduced pressure. The crude was purified by chromatographic column using AcOEt/acetone 9/1 as eluent system. Amorphous solid (55%). ^1^H-NMR (400 MHz, CD_3_OD) *δ* 3.19–3.21 (m, 1H, H-2); 3.48–3.54 (m, 4H, H-1, H-3); 7.04–7.09 (m, 5H, aryl); 7.40–7.43 (m, 2H, aryl); 7.84–7.89 (m, 2H, aryl). ^13^C NMR (100 MHz, CD_3_OD) *δ* 56.7 (C-2), 60.9 (C-1, C-3), 117.4, 117.7, 120.0, 121.4, 129.2, 130.1, 132.3, 151.1, 159.2(aryl). ESI-MS calc for C_15_H_17_NO_5_S 323.08; found 323.16. 

### 16.6. N-(1-Hydroxy-3-mercaptopropan-2-yl)-4-phenoxybenzenesulfonamide **(25d)**

To a 25 mL round-bottom flask, 2-amino-3-(tritylthio)propan-1-ol (3 mmol) were added and dissolved in DMF (10 mL). Cs_2_CO_3_ (1.5 eq.) and **24** (1.2 eq.) were then added, and the reaction was stirred for 24 h at room temperature. The reaction mixtures were washed with water, dried with Na_2_SO_4_, and evaporated under reduced pressure. The compound N-(1-hydroxy-3-(tritylthio)propan-2-yl)-4-phenoxybenzenesulfonamide was purified by chromatographic column using *n*-hexane/AcOEt: 2/1. Yield: 42%. ^1^H-NMR (400 MHz, CDCl_3_) *δ* 2.34 (d, *J* = 8.0 Hz, 2H, H-3); 3.18–3.23 (m, 1H, H-2); 3.42–3.49 (m, 2H, H-1); 7.14–7.70 (m, 24H, aryl); 7.74 (d, 2H, aryl). This intermediate was then dissolved in a 1 : 1 mixture DCM/TFA (10 mL), adding triethylsilane (0.1 eq) as scavenger. The reaction was stirred for 2 h at room temperature and evaporated under reduced pressure to afford the title compound as an amorphous solid. Yield: 92%. ^1^H-NMR (400 MHz, CDCl_3_) *δ* 2.65–2.70 (m, 2H, H-3); 3.35–3.40 (m, 1H, H-2); 3.64–3.79 (m, 2H, H-1); 7.03–7.09 (m, 5H, aryl); 7.37 (d, *J* = 6.8 Hz, 2H, aryl); 7.84 (d, 2H, aryl). ^13^C NMR (100 MHz, CDCl_3_) *δ* 26.6 (C-3), 51.1 (C-1), 62.9 (C-3), 118.0, 121.0, 129.6, 130.5, 138.2, 152.0, 160.1(aryl). ESI-MS calc for C_15_H_17_NO_4_S_2_ 339.06; found 339.12.

### 16.7. N-(1-Hydroxy-3-(1H-imidazol-4-yl)propan-2-yl)-4-phenoxybenzenesulfonamide Hydrochloride **(25e)**

To a 25 mL round-bottom flask, **1e** (3 mmol) was added and dissolved in DMF (10 mL). Cs_2_CO_3_ (1.5 eq.) and **24** (1.2 eq.) were then added, and the reaction was stirred for 24 h at room temperature. The reaction mixture was then washed with water, dried with Na_2_SO_4_, and evaporated under reduced pressure. The product was precipitated from the crude with dry HCl/eter solution and the filtered washed with Et_2_O. White solid (51%) 241–243°C. ^1^H-NMR (400 MHz, CD_3_OD) *δ* 2.78–2.85 (m, 2H, H-3); 3.47–3.52 (m, 2H, H-1, H-2); 3.68–3.71 (m, 1H, H-1); *δ* 7.02–7.10 (m, 5H, aryl); 7.39 (d, *J* = 8.8 Hz, 2H, aryl); 7.48 (s, 1H, imidazole); 7.80 (d, 2H, aryl); 8.85 (s, 1H, imidazole). ^13^C NMR (100 MHz, CD_3_OD) *δ* 28.4 (C-3), 52.6 (C-1), 63.2 (C-3), 114.0, 117.8, 118.2, 120.10, 121.4, 129.2, 130.1, 133.2, 139.8, 151.1, 159.2 (aryl). ESI-MS calc for C_18_H_20_ClN_3_O_4_S 2409.89, found 409.91. 

## 17. Molecular Modeling

All calculations were performed on a DELL T5500 workstation, equipped with two Intel Xeon E5630 2.53 GHz processors. 

All compounds were manually built in Maestro version 9.3.5, [35] exploiting the Built facility and minimized to a derivative convergence of 0.001 kJÅ^−1^ mol^−1^, using the Truncated Newton Conjugate Gradient (TNCG) minimization algorithm, the OPLS2005 force field, and the GB/SA water solvation model implemented in MacroModel version 9.9 [23].

Conformational searches, applying the mixed torsional/low-mode sampling and the automatic setup protocol, were carried out on all minimized ligand structures to obtain the global minimum geometry of each molecule, to be used as the starting conformation for docking calculations with Glide, version 5.8 [24, 36, 37].

Three-dimensional coordinates of MMP-1, -2, -8, and -9 were downloaded from the Brookhaven Protein Data Bank [38] (PBD ID: 1HFC, 1QIB, 1I76, and 1GKC, resp.). Each 3D structure was submitted to the Protein Preparation routine in Maestro that allows fixing of receptor structures, eliminating water molecules and possible ligands, fixing bond orders, adding hydrogen atoms, and ionizing charged residues. Hydrogen bond network is optimized, and for each structure, a brief relaxation was performed using an all-atom constrained minimization carried out with the Impact Refinement module version 5.8 and the OPLS-2005 force field to reduce steric clashes that may exist in the original PDB structures. The minimization was terminated when the energy converged or the root mean square deviation (RMSD) reached a maximum cut-off of 0.30 Å. 

Glide energy grid was generated using the crystallographic ligand of 1I76 as the centre of the grid, after superimposing all MMPs structures under study. The size of the box was determined automatically on the basis of the ligand dimensions. The global minimum geometry of ligands was submitted to docking calculations in the previously prepared proteins. The van der Waals radii for nonpolar ligand atoms were scaled by a factor of 0.8, thereby decreasing penalties for close contacts. Receptor atoms were not scaled. A first docking run was carried out applying the Standard Precision settings of Glide. Ten poses were saved and resubmitted to docking with the Extra Precision (XP) settings; [39] one pose was saved in this second run. The best ranking pose for each ligand in each protein was submitted to Liaison [29] to derive the scoring function applying the LIE method. Ligands and receptors structures were minimized in free and bound states through 1000 TNCG steps, allowing receptor residues 15 A far from the ligand to be freely relaxed. Implicit GB/SA solvent model was applied for solvation energy calculation.

The calculated *U*_vdW_, *U*_ele_, and *U*_cav_ parameters were correlated to experimental activity data using Strike [30] and the Multiple Linear Regression method, validating the model through leave-one-out (LOO) cross-validation analysis. 

## Figures and Tables

**Table 1:** Synthesized ZBGs library.

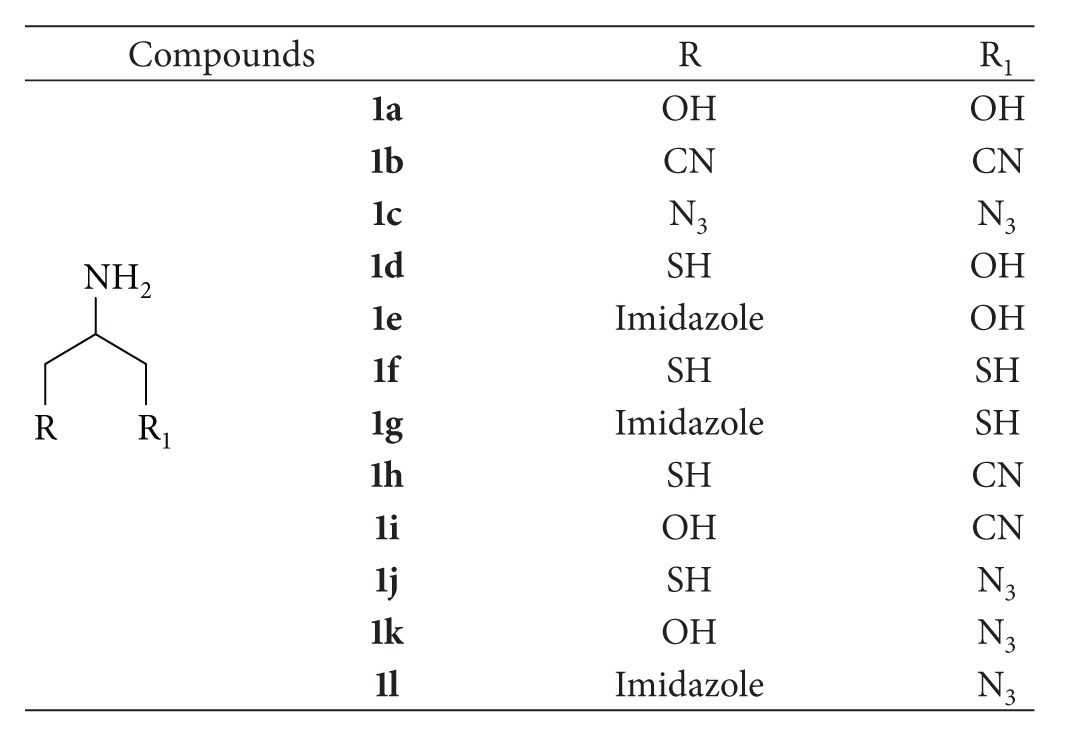

**Table 2:** Study on ditosylation reactionof N-Boc-serinol (**2**).

Entry	Solvents	Reaction time	TsCl : TEA	Yields (%)	Mono/di ratio
**1**	Py	6 h	2.4 : 3	29	10 : 1
**2**	Py	10 h	3 : 4	40	10 : 1
**3**	Py, DMAP cat	6 h	3 : 4	31	10 : 1
**4**	DCM, Py cat	6 h	2.4 : 3	53	5 : 1
**5**	DCM, Py cat	10 h	3 : 4	45	5 : 1
**6**	DCM	6 h	3 : 4	55	1 : 1
**7**	dry DCM	10 h	3 : 4	68	1 : 2
**8**	dry DCM	10 h	2.4 : 3	85	1 : 19

**Table 3:** Enzymatic inhibition of MMP-2 by 2-amino-1,3-disubstituted derivatives **1a–1l**.

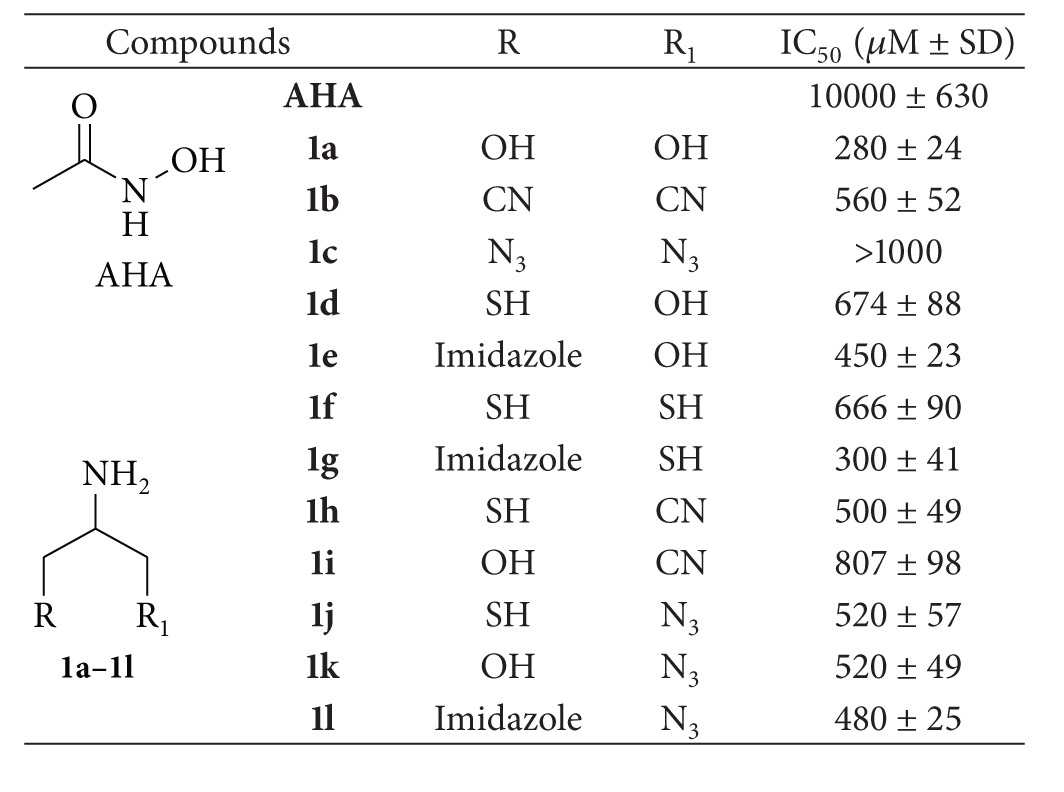

**Table 4:** Enzymatic activity of N-substituted phenoxybenzensulfonamide**25a**, **25d**, and **25e** on different MMPs.

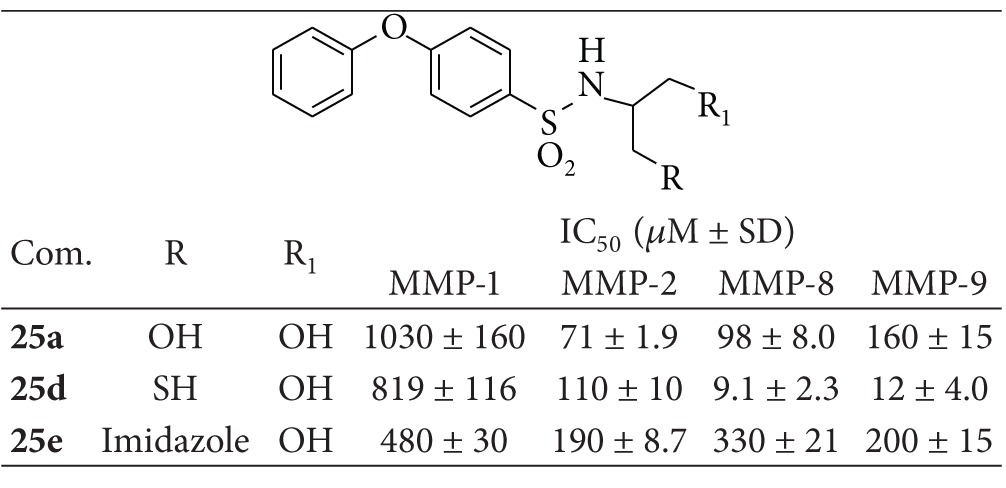

**Table 5:** Predicted and calculated binding energy (kcal) for all compounds toward MMP-2.

Compound	Δ*G* exp	Δ*G* calc (R-model)	Δ*G* calc (S-model)
**1a**	−4.837	−4.628	−4.484
**1b**	−4.428	−4.734	−4.619
**1c**	—^a^	—^a^	—^a^
**1d R**	−4.319	−4.512	
**1d S**			−4.492
**1e R**	−4.565	−4.395	
**1e S**			−4.614
**1f**	−4.333	−4.538	−4.592
**1g R**	−4.796	−4.583	
**1g S**			−4.739
**1h R**	−4.497	−4.421	
**1h S**			−4.303
**1i R**	−4.210	−4.391	
**1i S**			−4.379
**1j R**	−4.469	—^a^	—^a^
**1j S**			−4.511
**1k R**	−4.469	−4.237	
**1k S**			−4.513
**1l R**	−4.524	−4.624	
**1l S**			−4.338
**25a**	−5.655	−5.506	−5.326
**25d R**	−5.396	−5.349	
**25d S**			−5.279
**25e R**	−5.069	5.179	
**25e S**			−5.378

^a^No suitable docking poses were found.

**Table 6:** Statistical parameters for LIE R-model and S-model.

Model	*R* ^2^	SD	*F*	*P*	*R*_cv_^2^
R-model	0.813	0.218	13.1	0.00125	0.64
S-model	0.751	0.242	10.0	0.00232	0.511

**Table 7:** Predicted and calculated binding energy (kcal) for sulfonamide ligands toward MMP-1, -8, and -9.

Compound	MMP-1		MMP-8		MMP-9	
	Δ*G* exp	Δ*G* calc	Δ*G* exp	Δ*G* calc	Δ*G* exp	Δ*G* calc
**25a**	−4.074	−4.098	−5.460	−5.532	−5.178	−5.168
**25d R**	−4.224	−4.247	−6.870	−6.504	−6.705	−6.840
**25d S**		−4.176		−6.870		−6.528
**25e R**	−4.524	−4.527	−4.740	−4.365	−5.042	−4.970
**25e S**		−4.522		−5.408		−5.165
